# Catarrh of the Naso-Pharynx, or American Catarrh

**Published:** 1883-09

**Authors:** Morell Mackenzie


### Selections.

### Catarrh of the Naso-Pharynx, or American Catarrh.

[After defining the disease, and giving a short account of its very meagre literature, the lecturer remarked that postnasal catarrh was much more common in this country than was generally supposed, but that it was much milder than in America, where it was so universal that it might be looked upon as the national complaint. In referring to the cause of this affection, the lecturer gave the results of his recent observations in America in the following terms.]

Though I would not for a moment place my experience of American catarrh on a level with that of any of the eminent specialists who have given attention to the subject in the United States, I may remark that, in a recent tour through that country, I had a very favorable opportunity of studying the complaint; for I not only saw examples of the disease over a very wide tract of country, but also observed the atmospheric conditions under which these cases occurred—enjoying, moreover, the great advantage, in many localities, of discussing the subject and hearing the views of able physicians who had been studying the affection on the spot for many years. I was greatly astonished at the very wide diffusion of the affection. I met with it all over the Eastern States; it was very common in Chicago and St. Louis, which may now be called the central cities of America; I found it prevalent in Nebraska, and, to a slighter extent, in Utah; and, again, I encountered it on the Pacific coast, finding it very common in San Francisco. I had not the opportunity of seeing any patients in Nevada, as I merely traveled through that State without stopping; but in London I have treated many American travelers for postnasal catarrh, who had acquired the disease on the alkaline plains of the Silver State. I also saw a good many patients suffering from catarrh of the naso-pharynx in Colorado. In Southern California and Arizona, I scarcely met with any cases; and in Canada the affection, though much more common than in Europe, did not seem to be so universal as in the States. American catarrh, it would seem, principally prevails between latitude 44° and latitude 38°.

My travels in America were made in the latter end of August, and in September and October—that is, during the most favorable season of the year; and I have little doubt that, had I been there in the winter, I should have seen a great deal more of this wide-spread ailment. In many of the regions referred to, there are local conditions which tend to irritate the mucous membrane. Thus, all along the entire Eastern sea-board the atmosphere during the winter months is cold and moist, whilst in the summer it is excessively hot. In San Francisco, fogs prevail in the summer months in the early part of the day, whilst in the afternoon a cutting wind blows continuously. In Colorado, on the other hand, the climate is so extraordinarily dry, that only those who have been there can thoroughly appreciate it. The inhabited portion of the country consists of extensive plains situated at an elevation of 5,000 or 6,000 feet above the level of the sea. The dryness of the climate may be gathered from the fact that not a drop of rain falls during nine months of the year, the result being that no trees can flourish, the scrub oak being almost the sole representative* of our forest trees, and this being only found in the narrow valleys, or cañons, as they are called. Indeed, so extremely dry is the soil, that not unfrequently all the prairie-grass perishes. The atmospheric conditions, though admirably suited for some forms of consumption, are nevertheless extremely irritating to the mucous membrane of many persons. The white alkaline dust, which covers hundreds of miles in Nevada, is also met with here and there in Colorado. In the winter and spring, the winds are often rather strong, and it will easily be imagined that at auch times the abundant dust of this extraordinarily dry country is very irritating.

The soil of the American continent differs so widely in different parts, that it is impossible to suppose that it is concerned in the etiology of the affection. Again, it will be readily understood that the meteorological conditions over this vast area are so various, that they can not be regarded as a cause acting with anything like uniformity. The general character of the atmosphere of the American continent, as compared with that of Great

---

* The cotton-tree, though indigenous in certain parts of South America, appears to be an exotic in Colorado, and I only saw it as an ornamental tree in the streets and gardens of some of the cities.

Britain, and also with most parts of Europe, is that it is drier, that the changes of temperature are more sudden, and the extremes of temperature much greater. There is nothing, however, in these conditions to account for the localization of the complaint in the naso-pharynx; and it would seem that postnasal catarrh is not due to what may be strictly called climatic influence, but to something which is accidentally introduced into the atmosphere of widely differing localities; in other words, that there must be irritant particles floating in the air over very wide areas. This is actually the case, for *dust* is to be found everywhere in America.

The universal prevalence of catarrh is, indeed, fully explained by the abundance of dust, both in the country and in the cities. Owing to the immense size of the country, and its sparse rural population, the country roads have not, as a rule, been properly made, and, except in some of the older States, are merely the original prairie tracks. In the cities, notwithstanding the magnificence of the public buildings, the splendor of many of the private houses and the beauty of the parks, the pavement is generally worse than it is in the most neglected cities of Europe; such, indeed, as are only to be found in Spain or Turkey. It must be recollected also that, whilst in the decayed towns of the Old World there is very little movement, in the American cities there is a ceaseless activity and an abundance of traffic. Hence, the dust is set in motion in the one case, but not in the other. The character of the dust, of course, varies greatly, according to locality. In some parts, it is a fine sand, in others an alkaline powder; whilst in the cities it is made up of every conceivable abomination, among which, however, decomposing animal and vegetable matters are not the least irritating elements. An idea may, perhaps, be formed of the state of the atmosphere, from a consideration of the fact that in many cities the functions of the scavenger are quite unknown.

That a dusty atmosphere is the real cause of postnasal catarrh is rendered probable by a consideration of the anatomical relations of the naso-pharynx. For, owing to its being a *cul-de-sac* out of the direct line of the respiratory tract, particles of foreign matter which become accidentally lodged in its upper part are got rid of with difficulty—most likely by an increased secretion, which, as in the case of the conjunctiva, washes away any gritty substance which may temporarily alight on the membrane. As regards the larynx, irritating dust is expelled by coughing, which may be either reflex or voluntary; and again, in the case of the nasal passages, the minute particles of matter which constitute dust are expelled, if they happen to be obnoxious, either by sneezing or blowing the nose. But reflex acts, such as coughing and sneezing, have no effect on the upper part of the naso-pharynx, and it is only by a voluntary act, known as "hawking," that this cavity can be partially cleared. It is probable also that, owing to the sensibility of the naso-pharyngeal mucous membrane being less acute than that of either the nose or the larynx, minute foreign bodies accidentally lodged in the vault of the pharynx do not cause an amount of discomfort at all corresponding to that in the adjacent parts; hence, particles of matter are more likely to remain *in situ* for a long time time in the postnasal region than in either of the other parts, and are, of course, very apt to set up disease. In this country the complaint is most common in persons whose pharynx is large in the antero-posterior direction, a form of throat which facilitates the entrance, without favoring the expulsion, of foreign particles.

Whilst, however, it is highly probable that dust is the most frequent cause of postnasal catarrh, no doubt it is not the only one. Many circumstances favor its development. Some physicians have attributed it to the custom of over-heating houses by hot air and steam, as is commonly done in America. In the winter, the temperature is never allowed to fall below 70°, and it is generally much higher. The sudden passage from this temperature to that of the street is often not unlikely to set up catarrh; but as the same mode of heating is used in Russia without, as far as I am aware, giving rise to any postnasal affection, its influence can not be very great. The importance of heredity in the etiology of catarrh has been recently strongly insisted upon by Bresgen (*Der Chronische Nasen und Rachen-Katarrh*, Wien und Leipsig, 1883, p. 47); and, although no extensive series of exact observations have yet been made on this point, there is every probability that a disposition to catarrh may be inherited. I have seen so many instances, however, in which foreigners making a short stay in America have become affected with postnasal catarrh, that I think there is little doubt that atmospheric conditions—and those, let me add, of an accidental and controllable character— are much more powerful than heredity.—*Morell Mackenzie, M. D., in The British Medical Journal.*

